# Dual-Tunable Broadband Terahertz Absorber Based on a Hybrid Graphene-Dirac Semimetal Structure

**DOI:** 10.3390/mi11121096

**Published:** 2020-12-11

**Authors:** Jiali Wu, Xueguang Yuan, Yangan Zhang, Xin Yan, Xia Zhang

**Affiliations:** State Key Laboratory of Information Photonics and Optical Communications, Beijing University of Posts and Telecommunications, Beijing 100876, China; Flowing_Kinga@bupt.edu.cn (J.W.); yuanxg@bupt.edu.cn (X.Y.); xyan@bupt.edu.cn (X.Y.); xzhang@bupt.edu.cn (X.Z.)

**Keywords:** absorber, graphene, Dirac semimetal, dual-controlled, broadband

## Abstract

A dual-controlled tunable broadband terahertz absorber based on a hybrid graphene-Dirac semimetal structure is designed and studied. Owing to the flexible tunability of the surface conductivity of graphene and relative permittivity of Dirac semimetal, the absorption bandwidth can be tuned independently or jointly by shifting the Fermi energy through chemical doping or applying gate voltage. Under normal incidence, the device exhibits a high absorption larger than 90% over a broad range of 4.06–10.7 THz for both TE and TM polarizations. Moreover, the absorber is insensitive to incident angles, yielding a high absorption over 90% at a large incident angle of 60° and 70° for TE and TM modes, respectively. The structure shows great potential in miniaturized ultra-broadband terahertz absorbers and related applications.

## 1. Introduction

Terahertz (THz) absorbers have been widely investigated due to their wide applications in electromagnetic radiation, radiation, sensation, and thermal imaging [1,2,3]. A conventional terahertz absorber unit cell is typically composed of metallic materials, which can only operate at fixed predesigned frequencies with a relatively narrow absorption bandwidth [4,5]. In recent years, graphene and 3D bulk Dirac semimetal (BDS) are emerging as promising candidates for dynamically tunable broadband absorbers in the terahertz range [6]. Graphene, a new type of two-dimensional material, has attracted great interest due to its remarkable optical properties, high carrier mobility, and tunable surface conductivity within the terahertz frequency range [7]. Similarly, the relative permittivity of BDS, a material that can be considered as “3D graphene”, can also be dynamically controlled by an external gate voltage [8,9]. Up to now, some graphene- or Dirac-semimetal-based devices have been reported in the terahertz range [10,11,12,13,14,15,16,17,18]. Nevertheless, the bandwidth of these absorbers is not wide enough, and the tuning method is relatively monotonous. Therefore, it is worthwhile to develop advanced THz absorbers with much higher bandwidth and more flexible tuning ways.

In this work, a dual-controlled ultra-broadband THz absorber based on a hybrid graphene-Dirac semimetal multilayer structure is proposed and studied. By simply shifting the Fermi levels of graphene and Dirac semimetal, the proposed structure shows tunable absorption over a broadband wavelength range. The simulated results show that under normal incidence, the device exhibits a high absorption larger than 90% over a broad range of 4.06–10.7 THz for both TE and TM polarizations. Moreover, the absorber is insensitive to incident angles, yielding a high absorption over 90% at a large incident angle of 60° and 70° for TE and TM modes, respectively. The physical mechanisms are also elucidated by the impedance matching theory and electric field analyses.

## 2. Materials and Design

### 2.1. Graphene Material

**Figure 1:**
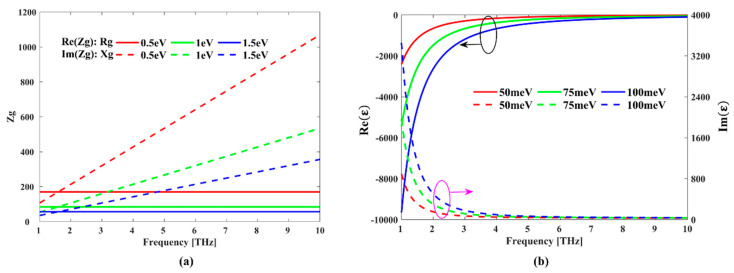
(**a**,**b**) Frequency-dependent surface impedance Zg of graphene and permittivity of bulk Dirac semimetal (BDS) at different Fermi energies, respectively.

It is known that graphene is a potential material to design reconfigurable THz devices due to the tunable surface conductivity $\sigma_{g}\left(\omega \right)$, which can be indicated by the Kubo’s formula [19]:

(1) $$\sigma_{g}\left(\omega \right)=\frac{e^{2}k_{B}T}{\pi \hbar^{2}}(\frac{\mu }{k_{B}T}+2\ln(e^{-\frac{\mu }{k_{B}T}}+1))\frac{-i}{\omega -i\tau^{-1}}+\frac{-ie^{2}}{4\pi \hbar }\ln(\frac{2|\mu |-(\omega -i\tau^{-1})\hbar }{2|\mu |+(\omega -i\tau^{-1})\hbar })$$

where μ is the Fermi energy (or chemical potential) of graphene which can be controlled by applying bias voltage or chemical doping, T is the temperature, i is the imaginary unit, e is the charge number of an electron, $k_{B}$ is Boltzmann’s constant, ħ=h/2π is the reduced Plank’s constant, and τ is the relaxation time. In the following simulations, we set the parameters as T=293 K and τ = 0.1 ps. From Equation (1), we can learn that the conductivity $\sigma_{g}\left(\omega \right)$ is related to frequency ω and Fermi energy μ adjusted by gate voltage. Furthermore, the surface impedance of graphene $Z_{g}$ is expressed as ${\;Z}_{g}=1/\sigma_{g}$,  Rg=Re(Zg), and  Xg=Im(Zg), where $R_{g}$ is the resistance of graphene and $X_{g}\;$is reactance of graphene. The frequency-dependent resistance $R_{g}$ and reactance $X_{g}$ are shown in Figure 1a. It can be found that at a fixed Fermi energy, the resistance $R_{g}$ keeps almost constant, while reactance $X_{g}$ increases linearly with increasing frequency.

### 2.2. BDS Material

In the THz range, the relative permittivity of BDS can be expressed as [20]:

(2) $$\varepsilon =\varepsilon_{b}+i\frac{\sigma }{\varepsilon_{0}\omega }$$

where $\varepsilon_{0}$ is the permittivity of vacuum and $\varepsilon_{b}=1$.

The dynamic conductivity *σ* can be written as [21]:

(3) Reσ=e2gkF24πħ(ħωEF+iħτ−1EF)θ(ħωEF+iħτ−1EF−2)

(4) Imσ=e2gkF24π2ħ[4ħωEF+iħτ−1EF−ħωEF+iħτ−1EFln(4εc2|(ħωEF+iħτ−1EF)2−4|)]

where ħ is the reduced Plank’s constant, $k_{F}=E_{F}/\hbar \nu_{F}$ is the Fermi momentum, $E_{F}$ is the Fermi energy applied to the BDS, $\nu_{F}{=10}^{6}{\;\mathrm{ms}}^{-1}$ is the Fermi velocity, $\tau =4.5\times 10^{-13}$,${\;g\;=\;40,\;\varepsilon }_{c}\;=\;3$, and θ(t) is the Riemann–Siegel theta function θ(t), which can be illustrated as [22]:

(5) $$\theta \left(t\right)=-\frac{i}{2}\left[\ln\Gamma \left(\frac{1}{4}+\frac{it}{2}\right)-\ln\Gamma \left(\frac{1}{4}-\frac{it}{2}\right)\right]-\frac{t\ln\left(\pi \right)}{2}$$

From Equations (2)–(5), we can see that the permittivity of BDS can also be controlled by Fermi energy. Figure 1b displays the frequency-dependent real and imaginary parts of BDS at different Fermi energies. It reveals that the real and imaginary parts of permittivity vary quickly in the range of 1–5 THz. The resonance frequency is mainly influenced by the real part of permittivity, while the loss is affected by the imaginary parts.

### 2.3. Design Method

**Figure 2:**
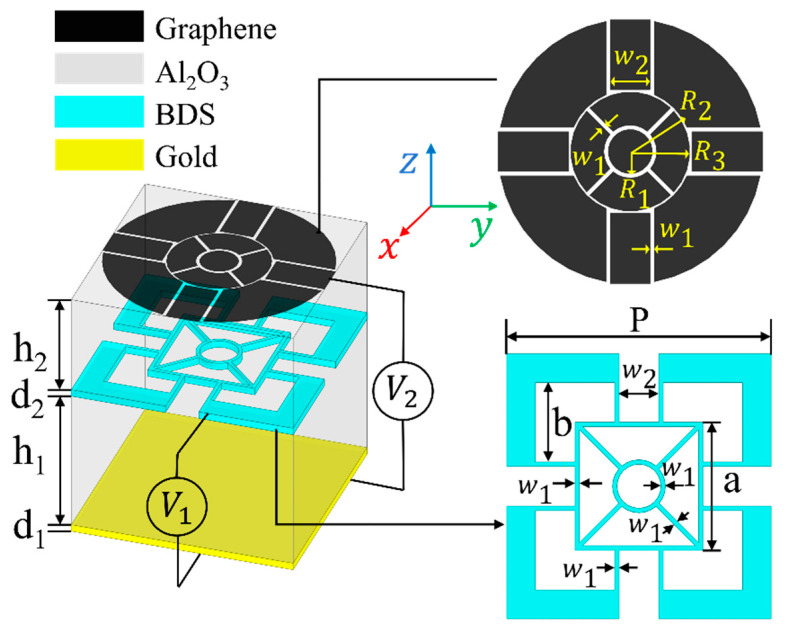
Schematic diagram of the proposed THz absorber.

The schematic diagram of the dual-controlled ultra-broadband THz absorber is illustrated in Figure 2. The structure is composed of five parts, which are top monolayer graphene pattern film, upper Al_2_O_3_ layer with a relative permittivity of 2.28 and loss tangent of 0.04, BDS complementary pattern layer, lower Al_2_O_3_ layer, and gold layer with conductivity σ = 4.56 × 10^7^ S/m from top to bottom. In this work, AlCuFe is used as the BDS material [16].

The top graphene patterned layer is to match the impedance of free space at a specific working frequency band, so as to achieve no reflection of the incident wave. The BDS pattern of the middle layer is to expand the absorption bandwidth. The basic function of the Al_2_O_3_ layer is to provide resonance absorption space, and the bottom gold layer is used to reflect the incident wave. In order to satisfy the polarization and incident angle insensitivity, we have designed a symmetrical structure as shown in Figure 2.

The reasons for designing this structure are as follows: (1) In the patterned graphene structure, due to the localized surface plasmon resonance, most of the electric field will be limited to the edge of the annular graphene. This resonance can effectively capture light energy to enhance the absorption rate. More circular channels in the graphene pattern can be arranged to limit the electric field distribution on the edge of the graphene, thereby increasing the absorption rate. Therefore, in our proposed structure, we use w_1_, w_2_, R_1_, R_2_, and R_3_ to construct circular rings and square channels in the graphene pattern. (2) In the design of the patterned BDS structure, on the one hand, the method of designing multiple small channels similar to the graphene layer is continued to be used; on the other hand, the structure is supposed to complement to the upper graphene pattern. In this case, the two absorption layers would resonate at different frequencies, thus extending the absorption bandwidth of the structure. In our proposed structure, the BDS pattern is controlled by w_1_, w_2_, a, and b. (3) In the conventional absorbing material model, in order to ensure that the wavelength in the dielectric layers forms a stable standing wave of electromagnetic waves, the thickness of the dielectric layer satisfies h = (2n + 1) λ/4, where *n* = 0, 1, 2.... In that case, the incident electromagnetic wave would interfere destructively with the reflected wave from the bottom metal layer, leading to the absorption of the electromagnetic wave energy by the structure. But for the metamaterial absorber, the thickness of the dielectric layer does not need to be a quarter wavelength as the dielectric constant and permeability of the metamaterial are controllable, which facilitates the realization of the absorber structure. In our work, the size of the two dielectric layers of graphene-BDS hybrid absorber is 4 μm and 2.8 μm, which are obtained from scanning optimization in the simulation software in consideration of the requirements of structural miniaturization and high absorption performance.

Ansys HFSS is used for the simulation of the proposed structure. The THz wave impinges on the graphene pattern from the air. Periodic linked boundary conditions (primary and secondary) are adopted in the x- and y-directions and Floquet port excitation in the z-direction. To better describe a graphene film, the graphene layer is considered as a two-dimensional conductive surface with the impedance boundary of resistance and reactance in the software simulations. For BDS material, the relative permittivity is created by importing relevant data obtained from Equations (2)–(5). Since the thickness of the bottom gold film is considerably larger than the skin depth, the electromagnetic waves can hardly be transmitted through the bottom metal plate, leading to S_21_ = 0 and absorbance A = 1 − |S_11_|^2^. The detailed dimensional parameters of the proposed absorber are listed in Table 1. These specific parameter values are generated by setting optimization goals that express as 1-(mag(S(FloquetPort1:1,FloquetPort1:1)))^2 > 0.9 in Ansys HFSS.

## 3. Results and Discussions

**Figure 3:**
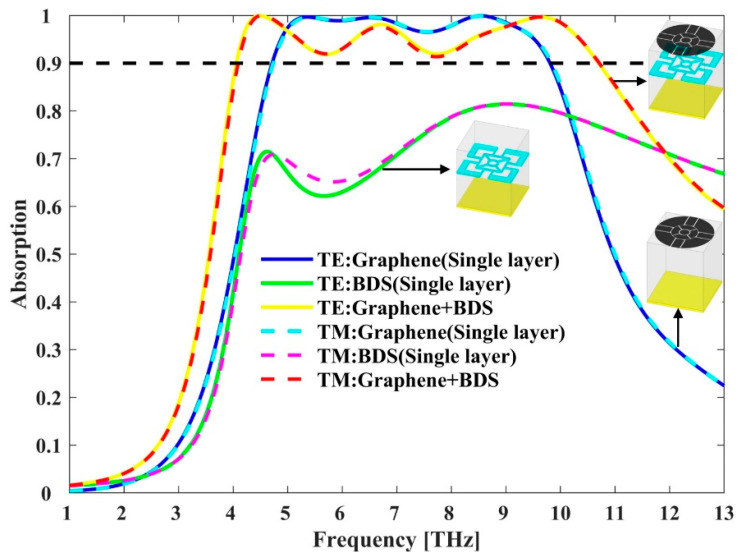
Absorption properties for different structures under normal incident TE and TM polarizations.

The absorption properties of different structures under normal incident TE and TM polarizations are shown in Figure 3. It can be seen that the BDS absorber exhibits a poor absorption lower than 90% over a broadband wavelength range of 1–13 THz. The graphene absorber shows high absorption above 90% from 4.72 THz to 9.83 THz, yielding an effective absorption bandwidth of 5.11 THz. By combining graphene and BDS, the absorption bandwidth is significantly increased. At a Fermi energy of 1.5 eV and 60 meV for graphene and BDS, respectively, the hybrid graphene-BDS structure exhibits effective absorption (above 90%) from 4.06 THz to 10.7 THz, yielding a much broader absorption bandwidth of 6.64 THz. To the best of our knowledge, the metamaterial absorber with an absorption bandwidth higher than 6.6 THz has not been reported yet. The center frequency $f_{c}$ is defined as $f_{c}=\left(f_{-}+\;f_{+}\right)/2=7.38\;\mathrm{THz}$, where $f_{-}$ and $f_{+}$ are the low- and high-frequency edges of 90% absorptance, respectively. Therefore, the fractional bandwidth (BW), the ratio of the absolute bandwidth to the center frequency is as high as 89.97%. To further illustrate the performance of the hybrid absorber, main parameters of reported THz absorbers based on graphene or BDS are listed in Table 2 for comparisons. Compared with other structures, the hybrid absorber exhibits significantly larger bandwidth and higher fractional BW with a small number of layers, showing great potential in miniaturized broadband THz applications.

**Figure 4:**
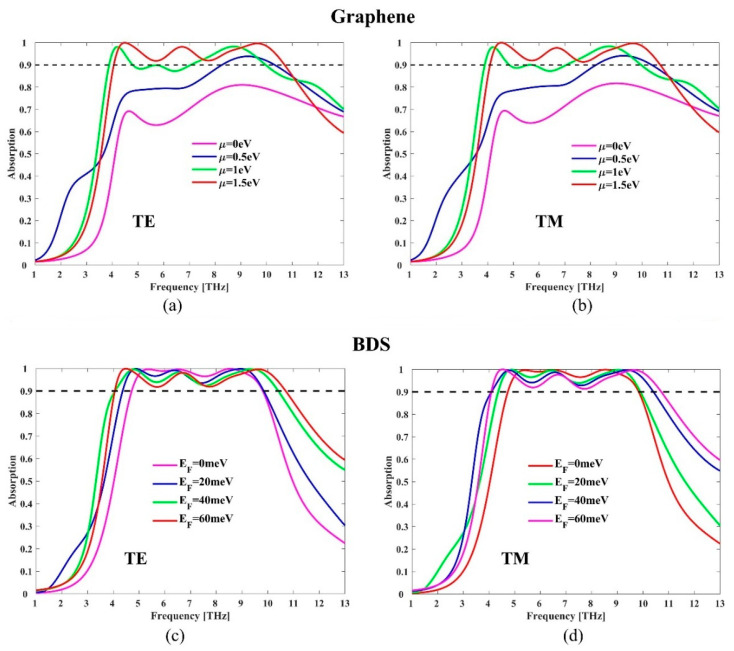
Absorption spectra with different graphene Fermi energy u = 0 eV, 0.5 eV, 1 eV, and 1.5 eV for (**a**) TE and (**b**) TM polarizations. Absorption spectra with different BDS Fermi energy E_F_ = 0 meV, 20 meV, 40 meV, and 60 meV for (**c**) TE and (**d**) TM polarizations.

Next, we studied the influence of graphene Fermi energy on absorption performance. Figure 4a,b display the absorption spectra of the proposed hybrid structure as a function of frequency and Fermi energy μ under normal incidence TE and TM polarizations, respectively. The Fermi energy $E_{F}$ of BDS is fixed at 60 meV. When the Fermi level of graphene μ is 0, only BDS functions as the absorption layer, and the effective absorption (above 90% absorption) is 0. As μ increases, the absorption gradually increases, and multiple absorption peaks gradually appear, leading to a significantly broader bandwidth. It can be learned from Equation (1) and Figure 1a that the conductivity $\sigma_{g}\left(\omega \right)$ and surface impedance in graphene are related with Fermi energy μ. As the Fermi level increases, the surface conductivity in graphene increases, and the plasma oscillation effect also increases, which ultimately leads to an increase in the absorption bandwidth. Therefore, the absorption performance of the hybrid structure can be effectively controlled by tuning the Fermi level of graphene in a broad wavelength range from 0 to 6.64 THz.

BDS offers another possibility to tune the absorption performance of the hybrid structures. Figure 4c,d present the absorption with different BDS Fermi energy $E_{F}$ of 0 meV, 20 meV, 40 meV, and 60 meV under normal incidence for TE and TM polarizations, respectively. The graphene Fermi energy μ is fixed at 1.5 eV. With the increase of the BDS Fermi level, the first absorption peak exhibits a redshift while the second and third absorption peaks show a blueshift, resulting in an increasing absorption bandwidth. It can be explained as follows: in the low terahertz band, the resonance frequency is mainly influenced by the real part of permittivity. It can be learned from Figure 1b that as the Fermi level increases, the real part of permittivity decreases, which leads to the decrease of resonance frequency and redshift of the absorption peak. In the high terahertz frequency band, the real part of BDS permittivity is close to 0 in Figure 1b. In this case, the resonance frequency is mainly affected by the localized plasmon resonance effect. As the Fermi level increases, the carrier density increases, leading to an increase of the plasmon resonance frequency and blueshift of the absorption peak. Thus, it can be concluded that the absorption performance of the hybrid absorber can be effectively regulated by means of tuning the resonance absorption peaks.

To sum up, owing to the tunability of absorption bandwidth by graphene Fermi energy and the control of resonance absorption peaks by BDS Fermi energy, our proposed absorber combines the advantages of being broadband-controlled and frequency-adjusted.

**Figure 5:**
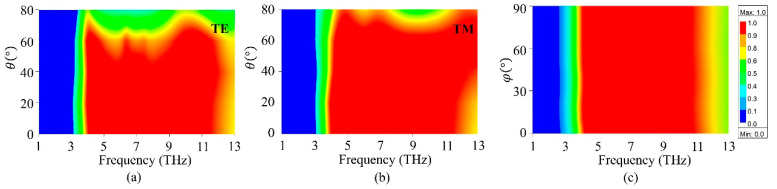
(**a**,**b**) Absorption maps as a function of incident angles θ for TE and TM polarization, respectively. (**c**) The absorption map for various polarization angles φ under normal incidence.

For THz absorbers, the sensitivity of polarization and incident angle is a significant issue in practical applications. Here, the absorption properties of the proposed structure under different polarization waves and incident angles are investigated, as shown in Figure 4. Here, the Fermi energy μ and EF of graphene and BDS are fixed at 1.5 eV and 60 meV, respectively. As shown in Figure 5a,b, the absorbance is almost independent of the incident angle up to 50° and 60° for TE and TM modes, respectively. For TE, the absorptance remains over 80% when the incident angle is θ up to 60°. With increasing incident angle θ, the absorptance gradually decreases and the absorption frequency is slightly expanded to a higher position. For TM polarized wave, the absorption is higher than 80% for incident angle up to 70°. However, the absorptance bandwidth slightly increases when θ becomes larger. Hence, the proposed absorber can tolerate a wide incident angle for both TE and TM polarizations. Figure 5c depicts the absorption spectra under normal incidence with different polarization angles φ. It can be seen that the absorptance is insensitive to the polarization angle, which is attributed to the symmetry of the structure. Therefore, the proposed structure can be utilized as a polarization- and incident angle-insensitive broadband THz absorber.

**Figure 6:**
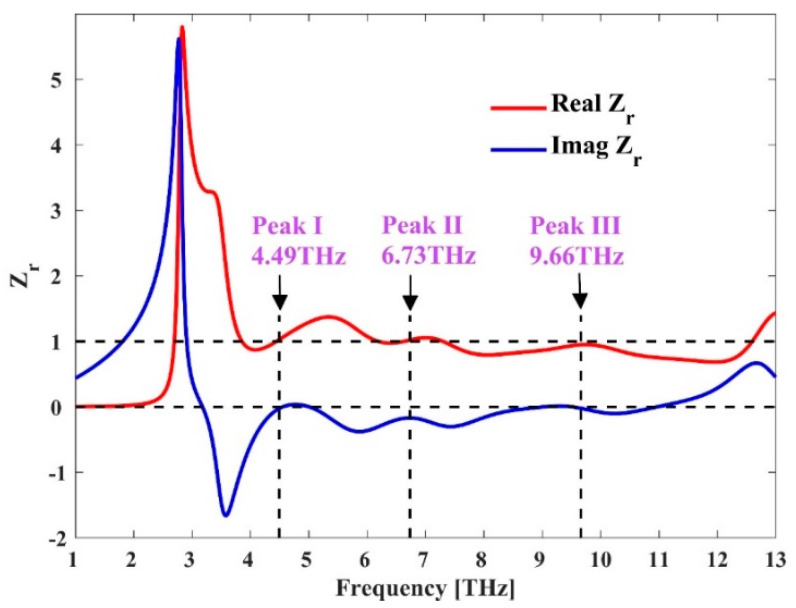
Real and imaginary parts of the relative impedance Z_r_.

To clarify the working principle, the absorption peaks located at 4.49, 6.738, and 9.66 THz are explained according to the impedance matching theory. The relative impedance (Zr) is calculated using Equations (6)–(10), where *d* is the travelled distance and *k*_0_ is the propagation constant of the wave at free space. The calculated values of the constitutive parameters for three absorption peaks are listed in Table 3. At all three frequencies, the real and imaginary parts of relative impedance Zr are very close to 1 and 0, respectively. More generally, Figure 6 presents the real and imaginary parts of the relative impedance for TE polarized wave in full terahertz band. We can see that in the frequency range from 4.58 THz to 10 THz, the real and imaginary parts of relative impedance are close to 1 and 0, respectively, which means the impedance of the absorber matches well with the free space, resulting in a broad absorption bandwidth.

(6) $$\chi_{es}=\frac{2i}{k_{0}}\frac{1-S_{11}}{1+S_{11}}$$

(7) $$\chi_{ms}=\frac{2i}{k_{0}}\frac{1+S_{11}}{1-S_{11}}$$

(8) $$\varepsilon_{eff}=1+\frac{\chi_{es}}{d}$$

(9) $$\mu_{eff}=1+\frac{\chi_{ms}}{d}$$

(10) $$Z_{r}=\sqrt{\frac{\mu_{eff}}{\varepsilon_{eff}}}$$

**Figure 7:**
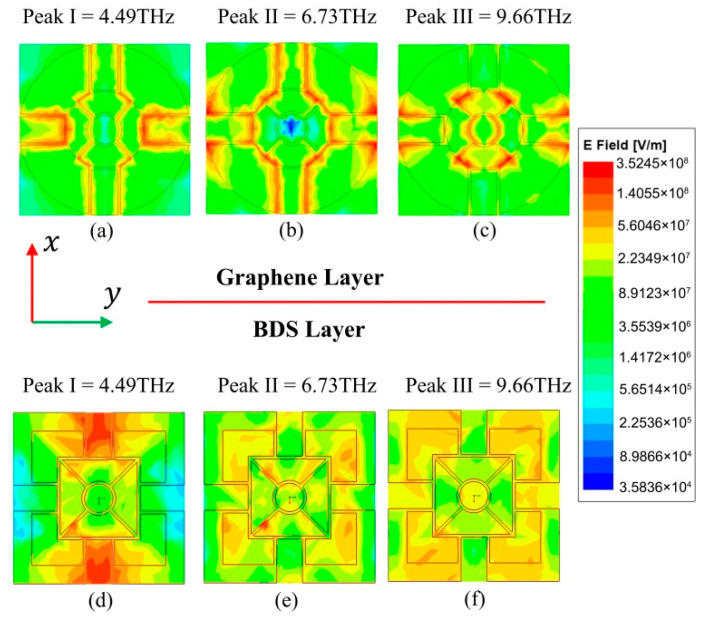
Absolute electric field distributions of graphene at (**a**) 4.49 THz, (**b**) 6.73 THz, and (**c**) 9.66 THz. Absolute electric field distributions of BDS at (**d**) 4.49 THz, (**e**) 6.73 THz, and (**f**) 9.66 THz.

In order to illustrate the working principle more intuitively, the electric field distributions on the x-y plane at frequencies of 4.49, 6.73, and 9.66 THz corresponding to absorption peaks I-III for TE polarization are shown in Figure 7. Figure 7a–c show the two-dimensional electric-field profiles of the graphene layer at the three frequencies, while Figure 7d–f present the two-dimensional electric field distribution of the BDS layer. At 4.49 THz, the electric field distribution of the graphene layer is mainly concentrated in the narrow gaps along the *x* and *y* directions and the internal oblique gaps, while the electric field distribution of the BDS layer is mainly concentrated in the wide channels along the *x* direction. At 6.73 THz, the electric field of the graphene layer is concentrated in each slit except for the four internal oblique slits, while the electric field of the BDS layer is scattered in the square blank channels. At 9.66 THz, the electric field of the graphene layer is concentrated in each slit except for the four slits in the *x* direction, and the electric field of the BDS layer is concentrated on the four sides of the *x* direction of the outer frame.

On the whole, as the frequency increases, the intensity of the electric field first increases and then decreases as the frequency increases in the graphene layer. As for the BDS layer, the change trend is opposite and firstly decreases and then increases. This is because that the incident wave excites carriers to oscillate along the *x*-axis and induce tangential electric fields on both graphene and BDS layers, which will cause energy loss. The energy consumption inside the loss materials including graphene, BDS and Al_2_O_3_ can be calculated by:

(11) $$A\left(f\right)=2\pi f\varepsilon^{\prime \prime }\int_{v}{\left|E_{l}\right|}^{2}dV$$

where $\varepsilon^{\prime \prime }$ is the imaginary part of dielectric constant, *V* is the volume of lossy material, and $E_{l}$ is the electric field inside the lossy materials. In the range of 1–13 THz, the imaginary parts of graphene and BDS are large. Therefore, the electromagnetic energy of THz wave will be dissipated where the electric field is strong.

**Figure 8:**
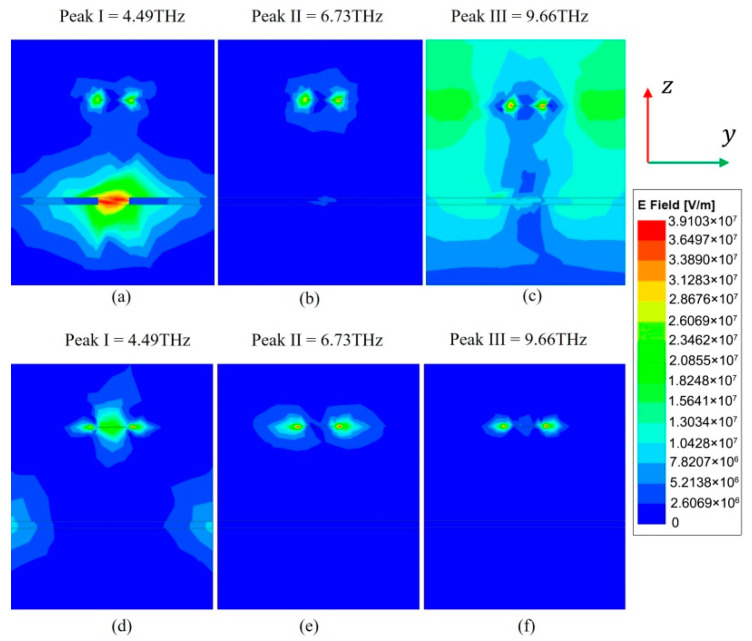
Absolute electric field distributions in the y-z planes to illustrate the energy localization at f = 4.49 THz, 6.73 THz, and 9.66 THz. (**a**–**c**) and (**d**–**f**) are the two adjacent faces in the structure.

**Figure 9:**
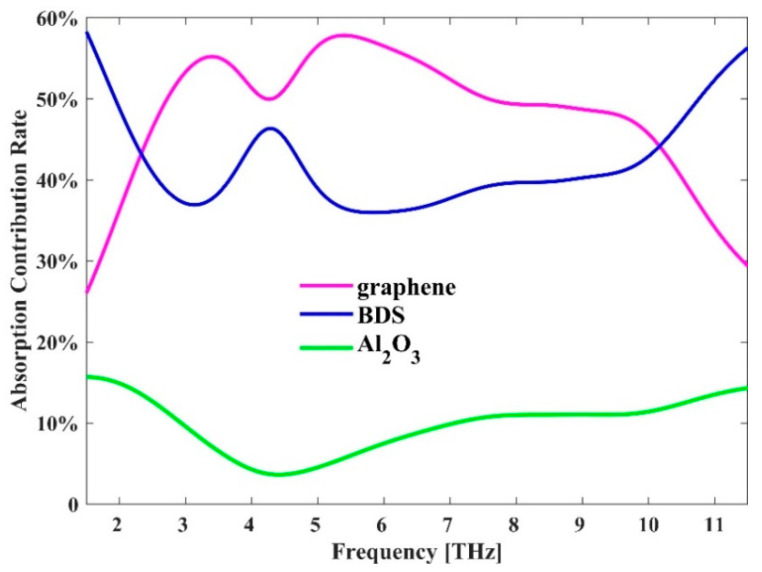
The absorption contribution rate of graphene, BDS and Al_2_O_3_ in the proposed hybrid absorber.

In order to understand how the energy is located at the resonance frequency, the electric field distribution in the y-z plane is simulated to reveal where absorption mainly occurs. Figure 8a–c show the cross-sectional views of the electric field |E|. It can be seen that the electric field is not only concentrated in different parts of the graphene and BDS patterns, but also trapped inside the dielectric layer, which means that the graphene pattern and BDS pattern, as well as the Al_2_O_3_ layer play an important role in absorption. The absorption contribution rate of each part is shown in Figure 9. We can see that the absorption in the graphene and BDS patterns is stronger than that in the Al_2_O_3_ layer, which is consistent with the absolute electric field distributions in Figure 7 and Figure 8. It could be mainly attributed to that the imaginary part of the dielectric constant of graphene and BDS is larger than that of the Al_2_O_3_ layer in the THz frequency range. Due to the symmetry of the structure, the TM polarization is the same as the TE polarization.

## 4. Conclusions

In summary, a tunable hybrid graphene-BDS broadband THz absorber is designed and studied. Due to the combination of advantages of graphene and BDS, the structure can achieve a high absorption exceeding 90% over a broad wavelength range of 6.64 THz. By adjusting the Fermi energy level of graphene and BDS, the absorption peaks and bandwidth can be dynamically tuned without reconstructing the structure. Besides, the absorber is insensitive to incident angles, yielding a high absorption over 90% at a large incident angle of 60° and 70° for TE and TM modes, respectively. Owing to its excellent performance, the proposed absorber has great potential in miniaturized ultra-broadband THz devices and microsystems.

## Figures and Tables

**Table 1:** Detailed dimensional parameters of the proposed absorber structure.

Parameter	Size (μm)	Parameter	Size (μm)	Parameter	Size (μm)
$d_{1}$	0.2	P	6	$R_{2}$	1.4
$d_{2}$	0.2	a	2.9	$R_{3}$	(a – 2 × $w_{1}$)/2
$h_{1}$	4	b	1.8	$w_{1}$	0.1
$h_{2}$	2.8	$R_{1}$	0.5	$w_{2}$	0.9

**Table 2:** Comparison of parameters of THz absorbers based on graphene or BDS.

References	Absorption Band (THz)	Fractional BW	Layers	Tunable Material	Polarization-Insensitivity	Angle-Insensitivity
[4]	3–7.8	88.8%	8	Graphene	insensitive	50°
[23]	5.50–9.10	8.2%	3	Graphene	insensitive	60°
[17]	<0.1	<5%	1	BDS	insensitive	60°
[24]	1.05–1.6	42.5%	2	Graphene and vanadium dioxide	insensitive	50°
This paper	4.06–10.7	89.97%	2	Graphene and BDS	insensitive	60°

**Table 3:** Calculations of relative impedance of the proposed structure at peak frequencies.

Frequency (THz)	Real Part of Zr	Imaginary Part of Zr
Peak I 4.49	1.027	−0.01
Peak II 6.73	1.021	−0.1673
Peak III 9.66	0.9495	−0.02
